# Mode of Delivery and Neonatal Transition: Insights from Electrical Cardiometry

**DOI:** 10.3390/children12020131

**Published:** 2025-01-26

**Authors:** Reem M. Soliman, Marwa M. Elgendy, Eman M. Metwalli, Zahraa Ezz ElDin, Antoine F. Abdel Massih, Hany Aly

**Affiliations:** 1 Department of Pediatrics, Cairo University, Cairo 11956, Egypt; mahmoudreem@rocketmail.com (R.M.S.); emanmetwalli29@gmai.com (E.M.M.); zahraezzeldin@yahoo.com (Z.E.E.); antoine.abdelmassih@kasralainy.edu.eg (A.F.A.M.); 2 Department of Pediatrics and Neonatology, University of Florida, Jacksonville, FL 32209, USA; 3 Department of Neonatology, Cleveland Clinic Children’s Hospital, Cleveland, OH 44195, USA; alyh@ccf.org

**Keywords:** heart rate, contractility, full-term infants, cardiac output, preterm, neonates

## Abstract

Background/Objectives: Examining hemodynamic changes during the early transition period aids in identifying variations in neonatal outcomes linked to ante- or intrapartum events. It facilitates the recognition of potential impacts stemming from common intrapartum management practices. The current literature provides scant insights into cardio-circulatory changes during the crucial first 10 min after birth. The application of Electrical Cardiometry (EC) emerges as a valuable noninvasive clinical tool for measuring neonatal hemodynamics. This prospective cohort study aimed to assess hemodynamic variables, including heart rate (HR), stroke volume index (SVI), index of contractility (ICON), and cardiac output index (COI) during the first hour of life in late preterm and full-term infants. Additionally, this study investigated the relationship between the mode of delivery and cardiovascular adaptation. Methods: Two hundred infants, encompassing both full-term and preterm, were enrolled, with categorization into four groups based on mode of delivery and gestational age. Hemodynamic variables were continuously evaluated using an EC device throughout the first hour of life. Findings: A significant decreasing trend was observed in HR, SVI, COI, and ICON over the first hour of life (*p* < 0.001). Infants delivered vaginally exhibited significantly higher HR, COI, SVI, and ICON compared to those born via Cesarean section (CS) (*p* = 0.006 and <0.001 and 0.035 and 0.001, respectively). Conclusions: This study highlights a consistent decreasing trend in HR, SVI, COI, and ICON over the first hour of life in both full-term and preterm infants. Notably, hemodynamic variables exhibited heightened levels in infants delivered vaginally compared to those born by CS.

## 1. Introduction

The transition from fetal to neonatal life is an intricate physiological transformation, necessitating significant adaptations in the respiratory and cardiovascular systems for successful extrauterine survival [1,2]. Various factors, such as maternal health, chronic medical conditions, placental status, gestational duration, and the presence of fetal anomalies, collectively regulate this intricate process. Initial steps in postnatal transition involve umbilical cord clamping, cessation of placental blood flow, clearance of fetal lung fluids, and tactile stimulation [3]. Clamping of the umbilical cord with cessation of placental blood flow, clearance of fetal lung fluids, and tactile stimulation are the initial steps for postnatal transition [4,5]. Hemodynamically, the transition entails a reduction in pulmonary vascular resistance as a result of lung inflation, coupled with an increase in systemic vascular resistance following cord clamping. While the majority of full-term neonates smoothly transition to extrauterine life without assistance, examining hemodynamic shifts during this early phase is essential for understanding neonatal outcomes related to antepartum or intrapartum factors, as well as typical intrapartum management practices [6,7]. The current literature lacks comprehensive reports on cardio-circulatory changes within the first 10 min after birth, emphasizing the need for detailed investigations [8,9]. Although echocardiography is the standard tool for assessing hemodynamic variables, its limitations in the delivery room, including the need for expertise, extensive training, and the inability to provide continuous cardiac output (CO) measurements, make it less practical. Additionally, echo may not always be readily available in the delivery room [10]. Electrical cardiometry (EC) emerges as a promising noninvasive clinical tool for neonatal hemodynamic monitoring. Validated for accuracy through concomitant measurements with echocardiography, EC can be conveniently performed on infants by utilizing four surface ECG electrodes, similar to cardiac monitor leads, making it suitable for delivery room use. Notably, there is a paucity of studies examining hemodynamic changes in late preterm infants or beyond 15 min of age in neonates using EC devices [8,9,10,11]. Understanding neonatal hemodynamic transitions contributes valuable insights for optimizing care in both late preterm and full-term infants during this critical period. This study aims to address the following: (a) Assessing the feasibility of utilizing an EC device for monitoring hemodynamics in the delivery room for both late preterm infants and full-term neonates. (b) Evaluating disparities in physiological transition in hemodynamics between late preterm infants and full-term neonates. (c) Examining the relationship between the mode of delivery and cardiovascular adaptation in neonates.

## 2. Material and Methods

### 2.1. Study Design

This prospective observational cohort study was conducted in the Obstetrics and Gynecology Department at Kasr El-Ainy, Cairo University Hospital. Before enrollment, written informed consent was obtained from the parents of the participants.

### 2.2. Inclusion Criteria

Two distinct groups of infants were enrolled in this study: a full-term newborn group with a gestational age (GA) of ≥37 0/7 weeks and a late preterm group with a GA of ≥34 0/7 to 36 6/7 weeks. Within each group, infants were categorized based on the mode of delivery, resulting in four subgroups: full-term vaginal delivery (FT VD), full-term Cesarean section (FT CS), late preterm vaginal delivery (PT VD), and late preterm Cesarean section (PT CS). Inclusion criteria comprised newborns delivered with Apgar scores of ≥7 at 1 min and ≥8 at 5 min, who successfully transitioned without the need for respiratory support or any resuscitation, adhering to the NRP resuscitation algorithm of the American Academy of Pediatrics.

### 2.3. Exclusion Criteria

Exclusion criteria encompassed neonates with any of the following conditions: (a) a history of fetal distress, demonstrated by fetal heart rate decelerations corresponding to a category 2 or higher heart rate tracing; (b) immediate need for active resuscitation, such as positive pressure ventilation, endotracheal intubation, and/or chest compressions at birth; (c) deliveries involving twins or multiple gestations; (d) neonates diagnosed with significant congenital anomalies, either confirmed through fetal ultrasound or detected upon initial post-birth examination; and (e) intrauterine growth restriction, defined by an estimated fetal weight below the 10th percentile. Additionally, neonates diagnosed with perinatal asphyxia during the study were excluded. Indicators of acute perinatal hypoxic–ischemic events included: (a) an Apgar score of less than 5 at 5 min; (b) fetal umbilical artery pH below 7.0 and/or base deficit of 16 mmol/L or greater; or (c) the presence of multisystem organ failure consistent with hypoxic–ischemic encephalopathy (HIE). Additional exclusions involved infants born to mothers with complications such as diabetes mellitus, hypertension, or clinical signs of chorioamnionitis. Infants born to mothers with a history of substance abuse or smoking were also excluded.

Descriptive data, including gestational age (GA), sex, and mode of delivery, were carefully recorded, with gestational age confirmed via prenatal ultrasound.

Electrical cardiometry monitoring system:

In this study, hemodynamic parameters were evaluated using the ICON C3 electrical cardiometry (EC) device, developed by Osypka Medical GmbH, Germany. Electrical cardiometry is a well-established, reliable, and non-invasive technique for monitoring hemodynamics in both children and infants. This method operates on impedance-based principles, which measure changes in the electrical impedance of the thoracic cavity as blood volume shifts with each heartbeat. The device employs four surface ECG electrodes, strategically placed on the skin to obtain accurate readings of cardiovascular function without the need for invasive procedures.

These electrodes were strategically placed on the left side of the forehead, left side of the neck, left hemithorax, and left thigh, while the transducer for arterial oxygen saturation and heart rate (HR) measurements was positioned on the right hand or wrist of the infants under study. The ICON electrical cardiometry system was employed to monitor cardiac output (CO) and other relevant hemodynamic parameters during the initial 15 min of life and up to one hour of age. Before commencing measurements, the skin was meticulously cleaned to remove vernix (caseosa), meconium, or blood, and the four surface ECG electrodes were affixed. Simultaneously, the transducer for arterial oxygen saturation and HR measurements was secured on the right hand or wrist. Monitoring was initiated between the first and second minute of life and continued until the fifteenth minute, with additional measurements recorded at the one-hour mark. ICON captured measurements for each parameter every 10 s, indicating the strength of the signal at each reading. Umbilical cord clamping occurred precisely 30 s after birth, with time accurately measured using a stopwatch. Neonatal resuscitation, performed by an attending neonatologist not affiliated with the research team, was administered when necessary. Apgar scores were documented at 1 and 5 min of life. Hemodynamic variables, including heart rate (HR), stroke volume index (SVI), index of contractility (ICON), cardiac output index (COI), thoracic fluid content (TFC), and oxygen saturation (SO_2_), were measured. All data were directly imported from the electrical cardiometry device. Continuous measurement and monitoring of newborns’ hemodynamics were executed for the entire first hour of life across all study groups.

### 2.4. Statistical Analysis

Data coding and entry were performed using the Statistical Package for the Social Sciences (SPSS) version 26 (IBM Corp., Armonk, NY, USA). For quantitative data, descriptive statistics such as mean and standard deviation were used, while categorical data were represented by frequency (count) and relative frequency (percentage). Group comparisons were conducted using the unpaired *t*-test or analysis of variance (ANOVA), ANOVA was used to compare the multiple measurements over time within each group, with post hoc testing applied as necessary. To assess changes over time within each group, repeated measures ANOVA was employed. Categorical data were analyzed using the Chi-square (χ^2^) test, with Fisher’s exact test applied when expected frequencies were below 5. The Pearson correlation coefficient was used to evaluate the relationship between continuous variables. A *p*-value of less than 0.05 was considered statistically significant. The primary aim of this statistical analysis was to test the assumption that CO, and other hemodynamic parameters would significantly decrease during the transitional period in term and preterm infants

### 2.5. Sample Size

Calculation of this study sample size was performed using the G-power Software, version 3.1.9.2. Calculation was based on neonatal cardiac output that was estimated to change from 206 mL/kg/min to 181 mL/kg/min [7]. To detect that change in CO a sample size of 82 infants would be required in each group (σ = 40 mL/kg/min, β = 0.2, and α = 0.05). To account for any attrition related to equipment failure or emergent need for clinical intervention/resuscitation, 18 infants (~20%) were added to each group. Therefore, 100 neonates would be recruited in each group (100 full term infants and 100 preterm infants).

## 3. Results

**Figure 1:**
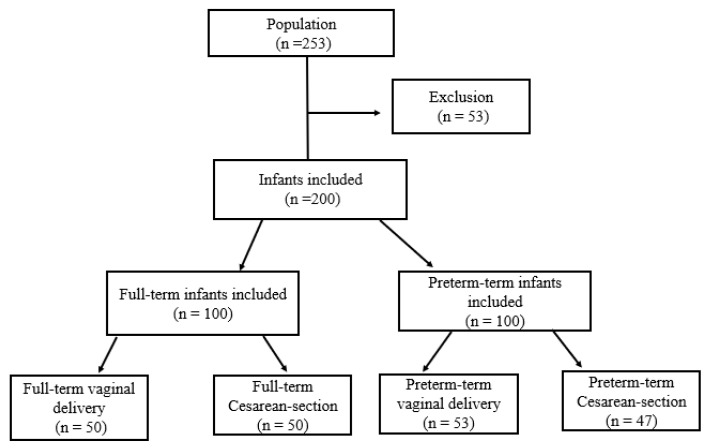
Flow diagram of the studied population.

Out of 253 newborns initially screened for this study, 53 were excluded due to factors such as the need for respiratory support, failure to obtain informed consent from parents, and equipment failure. Consequently, 200 neonates were successfully recruited, and their demographic characteristics were outlined in Table 1 and Figure 1.

**Figure 2:**
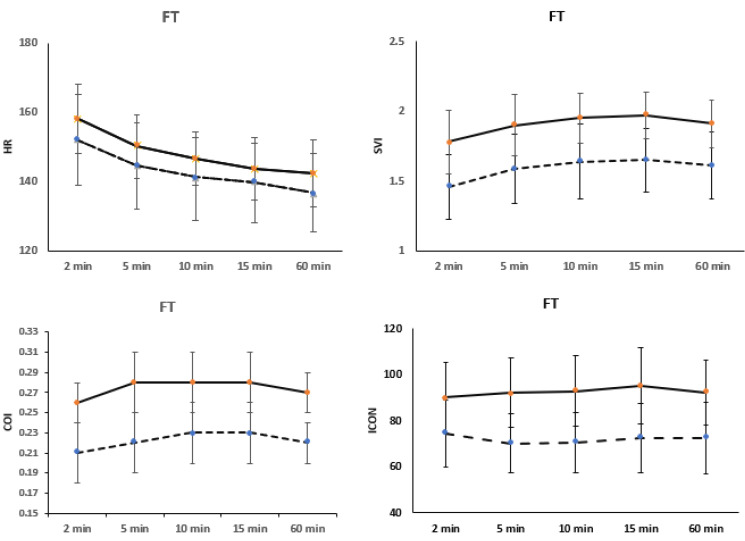
Changes in hemodynamic variables, such as cardiac output index (COI), stroke volume index (SVI), heart rate (HR), and index of contractility (ICON) during the first hour of life, are categorized by the mode of delivery in full-term infants (FT). The straight line represents vaginal delivery (VD), while the dotted line indicates full-term infants delivered by Cesarean section (CS).

Examining the heart rate (HR) trends among full-term infants delivered by vaginal delivery and C-section revealed a significant decrease at 2, 5, 10, and 60 min after birth (*p* = 0.014, 0.019, 0.021, and 0.006, respectively), as depicted in Figure 2. Furthermore, stroke volume index (SVI), cardiac output index (COI), and index of contractility (ICON) were found to be higher in full-term infants delivered vaginally compared to those delivered by C-section (*p* < 0.001). The trends for SVI, COI, and ICON displayed a notable increase over the first 60 min in both delivery modes (*p* < 0.001).

**Figure 3:**
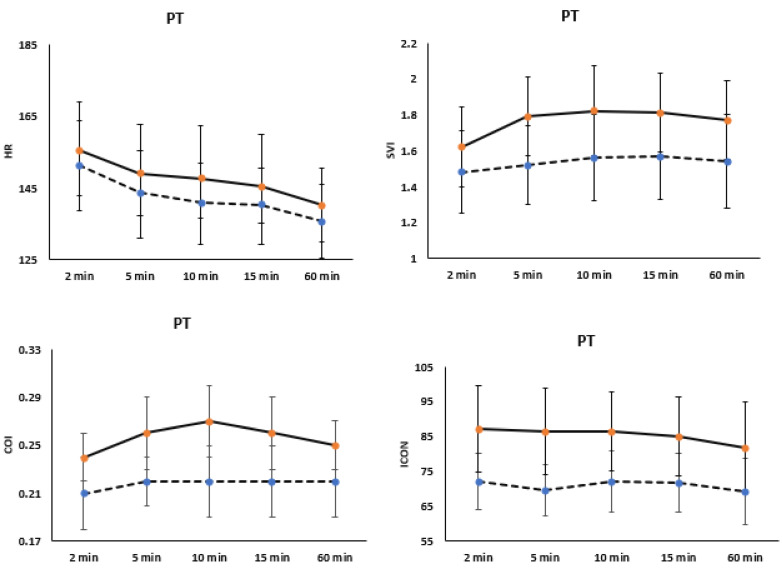
Changes in hemodynamic variables, such as cardiac output index (COI), stroke volume index (SVI), heart rate (HR), and index of contractility (ICON) during the first hour of life, are categorized by the mode of delivery in late preterm infants (PT). The straight line represents vaginal delivery (VD), while the dotted line indicates preterm infants delivered by Cesarean section (CS).

Analyzing hemodynamic variables among preterm infants revealed significant decreases in HR trends from the first 5 min of life to 60 min, irrespective of the delivery mode (*p* < 0.001). Similarly, SVI, COI, and ICON were higher in preterm infants delivered vaginally compared to those delivered by C-section (*p* < 0.001), with increasing trends over the first 60 min (*p* < 0.001) Figure 3.

The SVI showed a significant increase during the first 5 min of life in all groups of full-term infants. A notable increase in SVI was also observed between 5 and 10 min of life in the full-term vaginal (FT VD), and full-term cesarean section (FT CS) groups (*p* = 0.027, and 0.016, respectively). However, between 15 and 60 min of life, a significant decrease in SVI was noted in the FT VD (*p* = 0.001), as shown in Figure 2.

The cardiac output index (COI) increased during the first 5 min of life across all groups (*p* < 0.001). A significant increase in COI was observed between 5 and 10 min in full-term delivered by Cesarean section (*p* = 0.033). From 15 to 60 min of life, COI significantly decreased in full-term groups, (*p* = 0.036, and 0.04, respectively), as illustrated in Figure 2.

**Figure 4:**
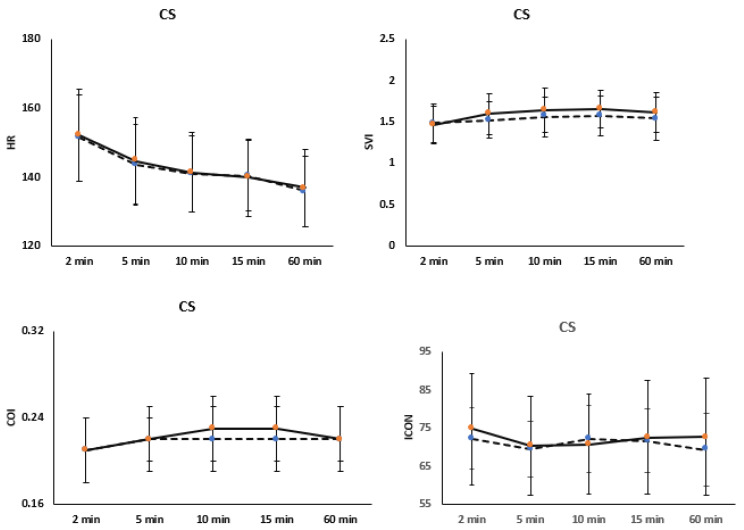
Trends of hemodynamic variables including cardiac output index (COI), stroke volume index (SVI), heart rate (HR), and index of contractility (ICON) during the first hour of life in both full-term and preterm infants who were delivered by C-section. The straight line represents full term infants delivered by C-section, while the dotted line indicates preterm infants.

**Figure 5:**
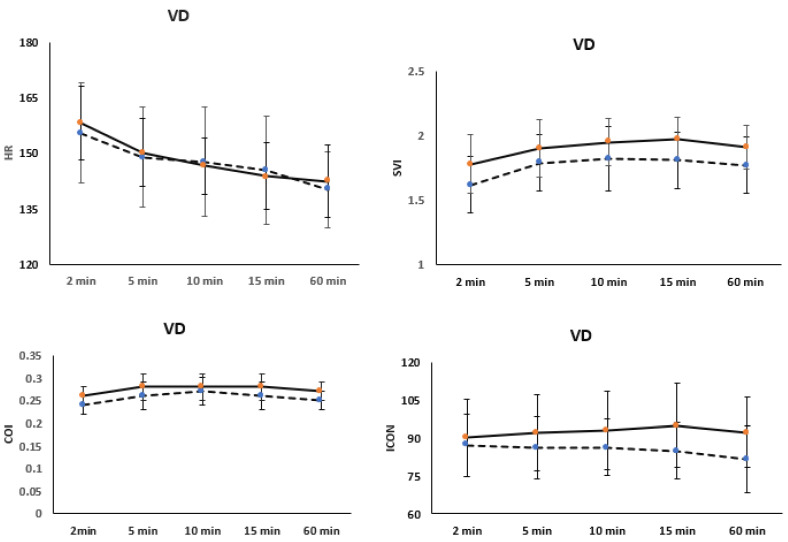
Trends of hemodynamic variables including heart rate (HR), stroke volume index (SVI), cardiac output index (COI), and index of contractility (ICON) during the first hour of life in both full-term and preterm infants who were delivered vaginally. The straight line represents full term infants delivered vaginally, while the dotted line indicates preterm infants.

Figure 4 and Figure 5 illustrate comparisons of hemodynamic variables among full-term and preterm infants based on the mode of delivery. In Figure 4, no significant changes are noted in HR, SVI, and ICON trends in both full-term and preterm infants delivered by C-section. In Figure 5, the analysis of hemodynamic variable trends among infants delivered vaginally showed no significant difference in HR trends over the first hour of life. However, SVI and COI differed significantly between full-term and preterm infants delivered vaginally, decreasing over time (*p* = 0.002 and p < 0.001, respectively). ICON trends exhibited a significant increase in full-term infants delivered vaginally compared to preterm infants (*p* < 0.001), showing a significant decrease over time starting from 15 min of life (*p* < 0.001).

## 4. Discussion

In this prospective study, the HR trend exhibited a significant decrease within the initial hour of life for both full-term and preterm infants. Meanwhile, the trends in SVI and COI showed a notable increase over time. The ICON trend saw a significant decline in both full-term and preterm infants throughout the first hour of life in infants who were delivered vaginally. When examining hemodynamic variables between full-term and preterm infants, HR did not reveal any notable difference, but SVI, COI, and ICON exhibited significant increases in full-term infants compared to their preterm counterparts. Interestingly, both full-term and preterm infants delivered vaginally displayed higher HR, SVI, COI, and ICON in comparison to those born via Cesarean section.

This study demonstrated the feasibility of utilizing the Electrical Cardiometry (EC) device to continuously monitor hemodynamic changes in real-time during the first hour of life in both full-term and preterm neonates. This is the first study to compare hemodynamic changes during the normal transition between full-term and preterm neonates, while also examining the impact of the mode of delivery on hemodynamic variables during this critical period using the EC device.

Continuous hemodynamic monitoring during the first hour of life in full-term and preterm neonates poses challenges due to the presence of persistent shunts and the intricate physiological changes occurring during the transition from fetal to neonatal circulation. Despite reports on neonatal transition using echocardiography, its application in the delivery room is constrained, as it is not only limited in utilization but also poses the risk of destabilizing an infant. Conducting a comprehensive cardiac assessment with echocardiography can be time-consuming. In addition, modes of delivery can have profound effects on neonatal cardiovascular dynamics, influencing factors such as cardiac output, pulmonary function, and the overall stability of the infant after birth. The choice of delivery mode must therefore consider maternal and fetal factors to minimize cardiovascular stress on the neonate and promote a smoother postnatal transition.

Our study did not show any significant differences in gestational age or Apgar scores among the infants. All infants included in this study did not require resuscitation and had good Apgar scores. The primary aim of our study was to assess the efficacy and feasibility of using the EC device to detect hemodynamic trends in hemodynamically stable neonates. Future prospective studies are needed to evaluate the feasibility of using the EC device in hemodynamically unstable neonates and those requiring resuscitation.

In the delivery room, we employed the EC device for real-time continuous assessment of hemodynamic variables during the first hour of life. A decrease in heart rate (HR) during the initial hour, coupled with a relatively higher stroke volume index (SVI), contributed to an elevated cardiac output index (COI) within the first 60 min in both preterm and full-term infants, regardless of the mode of delivery. While observing the SVI trend over the first hour of life, there was a significant decrease in SVI in full-term and preterm neonates from the first 15 min to 1 h of life. SV, representing the volume of blood pumped by the heart with each beat divided by body weight, serves as an EC indicator parameter for intravascular volume, while SVI is calculated by dividing the stroke volume by the body surface (BSA) are (SVI = SV/BSA).

The lower HR and a relatively decreased SVI observed during the initial 15 min to 1 h of life contributed to a lower COI. It is noteworthy that in the mature heart, cardiac output is increased by elevating both heart rate and stroke volume. However, neonates lack the ability to increase stroke volume, relying primarily on an increased heart rate to compensate for low cardiac output. Our findings align with a recent study utilizing bioreactance (BR) to investigate cardiac output in full-term neonates during the first 2 h of life. This study concluded a decrease in cardiac output among full-term infants from birth to 2 h of life [13]. We present the pioneering observation of a diminished cardiac output index (COI) among preterm infants within the first hour of life who delivered vaginally. Additionally, we conducted a comparative analysis of COI between full-term and preterm infants. Electrical cardiometry provides theoretical insights into cardiac contractility, elucidated by the parameter ICON, derived from the rate of change in thoracic impedance. Myocardial contractility, denoting the ventricle’s ability to contract, is typically represented as an ejection fraction in an echocardiogram. Our study revealed a reduction in ICON during the first hour of life in both full-term and preterm infants, irrespective of the mode of delivery. Notably, the ICON was lower in preterm infants compared to their full-term counterparts. The reduced contractility observed in preterm neonates may be linked to a lower number of cardiac muscle fibers and an underdeveloped myocardium [13]. Notably, upon examining hemodynamic variables between full-term and late preterm neonates, this study found no significant difference in heart rate (HR) during the first hour of life [14]. In contrast, stroke volume index (SVI), cardiac output index (COI), and index of contractility (ICON) exhibited a significant increase in full-term compared to preterm infants. The lack of a significant difference in heart rate (HR) may be attributed to the more advanced maturation of the autonomic nervous system (ANS) in neonates. Heart rate variability (HRV), a key indicator influenced by ANS development, is notably impacted by gestational age. As gestational age increases, HRV also rises, reflecting faster ANS maturation and enhanced cardiac neuroregulatory function, particularly with an increase in parasympathetic tone following birth [15]. While reports have explored differences in cardiac function among full-term infants delivered vaginally or by Cesarean section (CS) using echocardiography [16], there is currently a lack of studies utilizing electrical cardiometry (EC) to compare cardiac adaptation between infants born vaginally and those born by CS. This study is considered the first to utilize an EC device to compare hemodynamic variables between full-term and preterm infants delivered vaginally and those born by CS. Our findings revealed elevated heart rate (HR), stroke volume index (SVI), cardiac output index (COI), and index of contractility (ICON) in both preterm and full-term infants delivered vaginally, in contrast to those born via CS. Vaginal delivery is associated with increased fetal stress that stimulates increased catecholamines and adenosine release that directly increase fetal cardiac function [17,18].

This study establishes the feasibility of employing non-invasive cardiometry for continuous hemodynamic monitoring in both full-term and preterm infants. While previous research has successfully utilized this technology to monitor hemodynamic changes in full-term infants, these studies primarily focused on its feasibility in that specific population [9,13]. Notably, the current study extends this investigation to include the application of non-invasive cardiometry in preterm infants, providing valuable insights into its potential utility across diverse neonatal contexts. Our findings on the use of electrical cardiometry (EC) in the delivery room for monitoring hemodynamic trends in late preterm and full-term infants have shown promising results, with significant clinical implications for neonatal care. As a non-invasive and straightforward tool, EC reduces the need for more invasive procedures like arterial lines, which carry potential risks. It enables the early detection of hemodynamic instability, allowing clinicians to adjust interventions such as fluid resuscitation and medication administration with greater precision. By continuously monitoring key parameters in real time, EC can assess the effectiveness of resuscitation efforts and guide neonatal interventions. This is especially valuable for late preterm infants, who are more vulnerable to complications such as persistent pulmonary hypertension and impaired cardiac function. The early detection of these issues facilitates timely interventions, improving long-term outcomes. Ultimately, EC can enhance neonatal care by supporting early identification of instability, minimizing invasiveness, and enabling more personalized care strategies, leading to improved outcomes and more efficient practices for both late preterm and full-term infants.

### Strengths and Limitations

Our current investigation is the first prospective study to assess hemodynamics in both full-term and preterm infants comprehensively. It also specifically addresses the impact of the mode of delivery on hemodynamic variables during the first hour of life. However, certain limitations exist in this study. Notably, premature infants with a gestational age of less than 34 weeks or those with low birth weight were not included, necessitating future research to assess hemodynamic variables in this population. Our study did not include infants born to mothers with comorbidities or substance abuse. Future research should investigate the use of electrical cardiometry in such infants, as conditions like diabetes, preeclampsia, ectopic pregnancy, or substance abuse may potentially impact neonatal hemodynamics and outcomes. Furthermore, the duration of changes in heart rate (HR), stroke volume index (SVI), cardiac output index (COI), and index of contractility (ICON) during the transition remains unclear in this study. This study followed the trend of hemodynamics for the first hour only. Thus, the long-term implications of these findings have not yet been determined. Subsequent studies are imperative to determine the longevity of these observed hemodynamic changes.

## 5. Conclusions

This study highlights a decreasing trend in heart rate (HR), stroke volume index (SVI), cardiac output index (COI), and index of contractility (ICON) over the first hour of life in both full-term and preterm infants, with a notable and significant reduction in ICON observed in preterm infants compared to their full-term counterparts. Additionally, hemodynamic variables demonstrated an increase in infants delivered vaginally compared to those born by Cesarean section. The electrical cardiometry device is a valuable tool for continuously monitoring hemodynamic changes during the transition period in both full-term and late preterm infants, in the delivery room and the NICU after transfer. It facilitates the early detection of hemodynamic instability, enabling prompt interventions that could improve outcomes. To further elucidate systemic blood flow and the pathophysiology in preterm infants, future studies are warranted to evaluate hemodynamics during the transition in infants with a gestational age of less than 34 weeks.

## Figures and Tables

**Table 1:** Demographic characteristics of the studied full-term and preterm neonates [12].

**Full-Term Neonates**	**Cesarean Section (*n* = 50)**	**Vaginal** **(*n* = 50)**	***p*-Value**
**Gestational age at delivery (weeks)**	38.2 ± 1.0	38.3 ± 1.1	1
**Birth weight (kg)**	3.13 ± 0.41	2.97 ± 0.35	0.194
**Length (cm)**	48.5 ± 1.9	48.2 ± 2.0	1
**BSA (m^2^)**	0.21 ± 0.02	0.20 ± 0.01	0.114
Male (%) *	22 (44%)	31 (62%)	0.071
**Apgar at 1 min**	7 (7 to 8)	8 (7 to 8)	0.497
**Apgar 5 at min**	9 (9 to 9)	9 (9 to 9)	1
**Preterm Neonates**	**Cesarean section** **(*n* = 47)**	**Vaginal** **(*n* = 53)**	***p*** **-Value**
Gestational age at delivery (weeks)	35.1 ± 0.8	35.2 ± 0.8	1
Birth weight (kg)	2.63 ± 0.30	2.54 ± 0.35	1
Length (cm)	46.7 ± 2.1	45.8 ± 2.3	0.248
SA (m^2^)	0.19 ± 0.01	0.18 ± 0.01	0.68
Male (%) *	23 (48.9%)	31 (58.5%)	0.339
Apgar at 1min	8 (8 to 8)	8 (7 to 8)	1
Apgar at 5 min	9 (9 to 10)	9 (9 to 10)	1
Antenatal steroids *	11 (23.4%)	18 (34%)	0.246

* Chi-squared test for nominal and unpaired *t*-test for numerical data. Data were expressed as mean±SD for numerical data and number and percentage for qualitative data. Surface area **(SA);** kilogram **(Kg)**; centimeter **(Cm).**

## Data Availability

Data are contained within the article.
